# Supplementary material for: Causal interplay between lactose intolerance and gut microbiota: a combined bidirectional Mendelian randomization and in vivo validation study
Source: Front Nutr. 2026 Jun 1;13:1803337. doi: 10.3389/fnut.2026.1803337 (PMC13265576; doi:10.3389/fnut.2026.1803337)
Supplement: Supplementary file 1 [file Data_Sheet_1.zip › supplementary materials/Forward/leave one out cross validation/ebi-a-GCST90027552.finngen_R12_E4_LACTONAS.pdf]

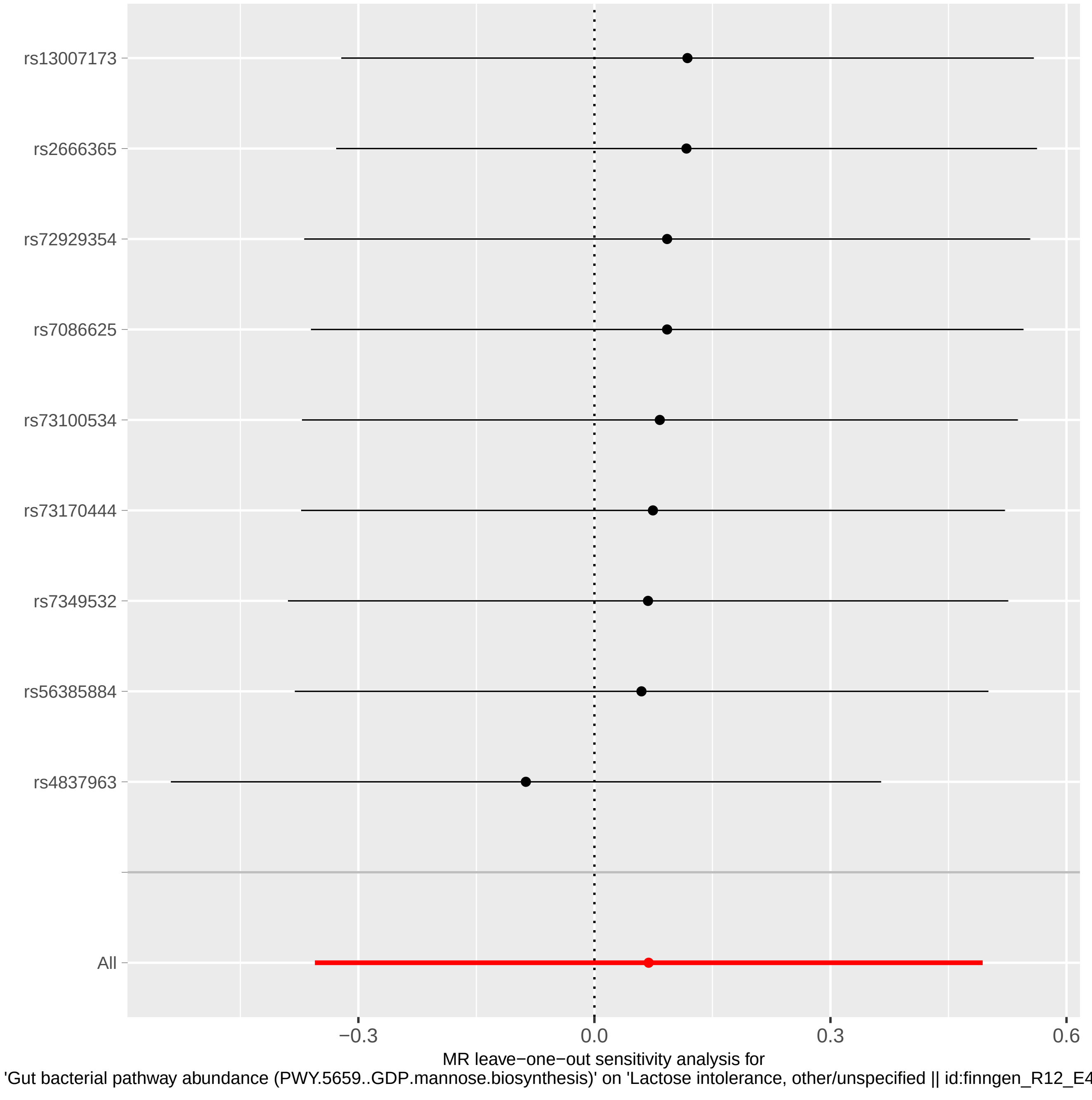
rs13007173
rs2666365
rs72929354
rs7086625
rs73100534
rs73170444
rs7349532
rs56385884
rs4837963
All
−0.3
0.0
0.3
0.6
MR leave−one−out sensitivity analysis for
'Gut bacterial pathway abundance (PWY.5659..GDP.mannose.biosynthesis)' on 'Lactose intolerance, other/unspecified || id:finngen_R12_E4
